# Dyslipidemia-induced renal fibrosis related to ferroptosis and endoplasmic reticulum stress

**DOI:** 10.1016/j.jlr.2024.100610

**Published:** 2024-07-31

**Authors:** Yamei Jiang, Xiangyang Zhu, Kyra Jordan, Yongxin Li, Sabena Conley, Hui Tang, Amir Lerman, Alfonso Eirin, Tongwen Ou, Lilach O. Lerman

**Affiliations:** 1 Division of Nephrology and Hypertension, Mayo Clinic, Rochester, MN, USA; 2 Department of Urology, Xuanwu Hospital, Capital Medical University, Beijing, China; 3 Department of Cardiovascular Diseases, Mayo Clinic, Rochester, MN, USA

**Keywords:** hypertriglyceridemia, hypercholesterolemia, ferroptosis, endoplasmic reticulum stress, renal fibrosis

## Abstract

Dyslipidemia may induce chronic kidney disease and trigger both ferroptosis and endoplasmic reticulum (ER) stress, but the instigating factors are incompletely understood. We tested the hypothesis that different models of dyslipidemia engage distinct kidney injury mechanisms. Wild-type (WT) or proprotein-convertase subtilisin/kexin type-9 (PCSK9)-gain-of-function (GOF) Ossabaw pigs were fed with a 6-month normal diet (ND) or high-fat diet (HFD) (n = 5–6 each). Renal function and fat deposition were studied in vivo using CT, and blood and kidney tissue studied ex-vivo for lipid profile, systemic and renal vein FFAs levels, and renal injury mechanisms including lipid peroxidation, ferroptosis, and ER stress. Compared with WT-ND pigs, both HFD and PCSK9-GOF elevated triglyceride levels, which were highest in WT-HFD, whereas total and LDL cholesterol levels rose only in PCSK9-GOF pigs, particularly in PCSK9-GOF/HFD. The HFD groups had worse kidney function than the ND groups. The WT-HFD kidneys retained more FFA than other groups, but all kidneys developed fibrosis. Furthermore, HFD-induced ferroptosis in WT-HFD indicated by increased free iron, lipid peroxidation, and decreased glutathione peroxidase-4 mRNA expression, while PCSK9-GOF induced ER stress with upregulated GRP94 and CHOP protein expression. In vitro, pig kidney epithelial cells treated with palmitic acid and oxidized LDL to mimic HFD and PCSK9-GOF showed similar trends to those observed in vivo. Taken together, HFD-induced hypertriglyceridemia promotes renal FFA retention and ferroptosis, whereas PCSK9-GOF–induced hypercholesterolemia elicits ER stress, both resulting in renal fibrosis. These observations suggest different targets for preventing and treating renal fibrosis in subjects with specific types of dyslipidemia.

## Graphical Abstract

**Figure:**
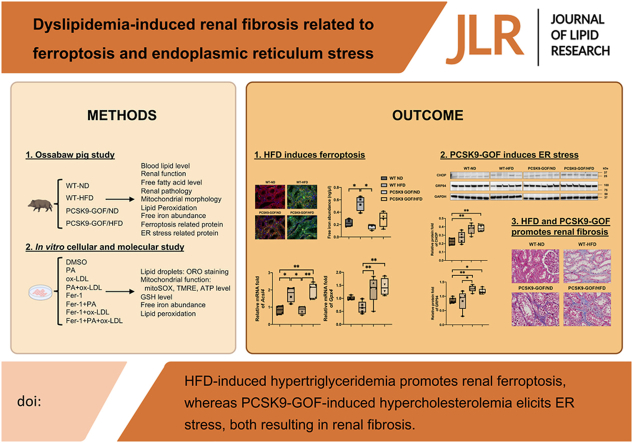

Metabolic syndrome manifests as obesity, insulin resistance, dyslipidemia, and hypertension, a cluster which is prevalent globally and significant clinically due to its close association with development of cardiovascular complications (1). Dyslipidemia—which involves disequilibrium of lipids such as triglycerides (TGs), total cholesterol, and LDL-C—is an extremely prevalent complex trait (2). In the United States, more than 100 million, or roughly 53% of adults, have elevated LDL-C levels (3). Yet, under 50% of patients received treatment to lower their LDL-C level and fewer than 35% achieved their control goal. (4).

Dyslipidemia is a well-recognized cause of atherosclerosis, cardiovascular diseases, and all-cause mortality (5, 6), and impacts the kidney as well. For example, C57BL/6 mice fed with high-fat diet (HFD) for 16 weeks have markedly increased kidney TG and cholesterol contents, with impaired renal function, increased oxidative stress, mitochondria fission, and activated apoptotic signaling (7). Similarly, we have previously shown that mitochondrial damage, increased production of reactive oxygen species (ROS), and oxidative stress characterize kidney alterations in HFD pigs (8, 9, 10). Furthermore, we have shown that Ossabaw pigs, which are prone to metabolic syndrome, developed renal adiposity with oxidative stress and inflammation, and that hyperfiltration correlated with tissue TG levels (11). In an extended model, Ossabaw pigs bearing a proprotein convertase subtilisin/kexin type-9 (PCSK9) gain-of-function (GOF) mutation and showing elevated LDL levels increased intra-renal fat deposition; we subsequently found renal oxidative stress and fibrosis, accompanied by peripheral arterial intimal thickening and atheromatous plaque development (12).

Lipid synthesis also occurs in the smooth endoplasmic reticulum (ER) (13), where ER stress could be accompanied by various pathological processes related to dyslipidemia. Non-alcoholic fatty liver disease due to HFD involves ER stress (14, 15), Furthermore, HFD-induced non-alcoholic steatohepatitis progressed to chronic kidney disease (CKD), characterized by ER stress, apoptosis, and renal fibrosis (16). Likewise, dyslipidemia and insulin resistance in obesity can lead to renal damage through the activation of inflammation, ER stress, apoptosis, and renal fibrosis (17).

Ferroptosis is a regulated cell death pathway characterized by iron-dependent, lipid peroxidation (LPO)-driven membranous structure destruction that is suppressible by glutathione peroxidase-4 (GPX4) (18). Acyl-CoA synthetase long-chain family member-4 (ACSL4), a ferroptosis regulator, catalyzes the incorporation of PUFAs into phospholipids to enhance ferroptosis (19). Abnormal lipid metabolism–induced ferroptosis is an important method of cell death, which in turn is involved in multiple complications of dyslipidemia. Stroke is often induced by hypertension and hyperlipidemia (20), and regulating ferroptosis has been identified in animal models as a new potential target for stroke (21). Additionally, inhibiting ferroptosis provides experimental support for emerging drug treatment of atherosclerosis and hyperlipidemia (22), and metformin-mediated anti-ferroptosis effects attenuate hyperlipidemia-associated vascular calcification (23). However, whether renal injury caused by dyslipidemia mechanistically involves ferroptosis has not been illustrated.

We tested the hypothesis that swine dyslipidemia activates ferroptosis. To this end, the present study investigated the renal function and injury processes in two different types of unique dyslipidemic Ossabaw pig models and explored their underlying mechanisms.

## Materials and Methods

### Animal model

This study was approved by the Mayo Clinic Institutional Animal Care and Use Committee. The PCSK9 GOF Ossabaw model was established as described previously (24). Briefly, the primate PCSK9 coding sequence with D374Y mutation (substituting an aspartic acid for tyrosine at amino acid 374) (Epoch Life Science, Missouri, TX) was introduced into male Ossabaw fibroblasts (Swine Resource, Indiana University, Bloomington, IN), which were subsequently used for cloning transgenic PCSK9-GOF founder line mini-pigs (Recombinetics, Saint Paul, MN).

Eleven wild-type (WT) and twelve PCSK9-GOF 3-month-old male Ossabaw mini-pigs were randomly divided into four groups: WT fed a normal diet (ND, n = 6) or HFD (n = 5), PCSK9-GOF-ND (n = 6), and PCSK9-GOF-HFD (n = 6). ND chow included 13% protein, 2% fat, 6% fiber, and 70% carbohydrate (nitrogen-free extract by subtraction), while HFD chow contained 19.8% fat (ether extract, with 2% cholesterol), 17% protein, 5.3% fiber, and 41.8% carbohydrate (Argi-Nutrition Service, Shakopee, MN).

### In vivo studies

After 6 months of feeding, animals were weighed and anesthetized with telazol (5 mg/kg) and xylazine (2 mg/kg). To measure serum creatinine (SCr) and lipid profile, a catheter was placed via the femoral vein to collect blood from the right and left renal vein (RV) and inferior vena cava (IVC), whereas urine was collected from the urinary bladder to measure albumin. Then, following a central venous injection of iopamidol (0.5 ml/kg over 2 s), multi-detector computed tomography (MDCT, Somatom Definition-128, Siemens, Forchheim, Germany) scanning was performed to evaluate their single-kidney function (glomerular filtration rate [GFR] and volume). Single-kidney GFR (mL/min/cc tissue) was calculated from the right and left cortical time-attenuation curves obtained from timed flow MDCT images. To assess intrarenal fat deposition in vivo, virtual non-contrast (VNC) maps and datasets were then generated from dual-energy MDCT images, as published before (25, 26).

### Ex vivo studies

#### Biochemical analysis and lipid profile

Animals were allowed to recover from the in vivo studies for 3 days, followed by euthanasia with intravenous pentobarbital (Fatal-Plus solution, 100 mg/kg, Vortech, Dearborn, MI) and kidney harvesting.

TGs, total cholesterol, LDL concentrations, and urinary albumin were determined following standard procedures. SCr was detected abide with manufacture’s instruction (K002-H, ARBOR ASSAYS). Nonesterified FFAs levels were measured in the blood from the RV and IVC using a mass spectrometer coupled with an ultra high performance liquid chromatography (LC/MS) system as previously described (27). Briefly, 50 ul of plasma was spiked with internal standard prior to extraction. The extracts were dried down and brought up in running buffer prior to injecting on the LC/MS. Data acquisition was performed under negative electrospray ionization condition. FFA profile included saturated fatty acids [SFAs: myristic acid, palmitic acid (PA), and stearic acid] and MUFAs (palmitoleic acid and oleic acid), PUFAs (EPA, linolenic acid, DHA, arachidonic acid, and linoleic acid], and trans fatty acid [TFA: elaidic acid]. Based on the assumption that the difference between infra-renal IVC and RV levels reflects FFA retention within the affected kidney (28), we estimated each renal FFA gradient (RV-IVC).

#### Histology and immunofluorescence

Kidney sections fixed with formalin were processed for Masson’s trichrome staining using standard techniques to assess the degree of interstitial fibrosis (IF) and tubular atrophy (TA). IF was determined in 10–15 fields of each kidney section in Masson’s trichrome stained slides using ImageJ (Fiji) following instructions. Firstly, we converted the image into RGB format, and then split color channels into three (red, green, and blue) channels, selected the blue channel, and adjusted its threshold to highlight the fibrotic areas. Next, we applied the threshold to obtain the percentage of fibrotic area for each image. Finally, the values of all fields were averaged to generate a single data point for each pig kidney for statistical analysis (29). TA was evaluated manually method in the same image set. The total numbers of tubules and atrophic tubules in each image were counted independently by two skilled investigators. The atrophic tubules were characterized by reduction in tubular epithelial cell size, decreased cytoplasm, shrunken or enlarged nuclei, and narrowed or completely obliterated tubular lumen. TA (%) was calculated as the number of atrophic tubules divided by the total number of tubules in each image average between the two investigators (30). Sections frozen in liquid nitrogen were sliced for Oil-red-O (ORO) and dihydroethidium staining (Thermo Fisher Scientific, Waltham, MA) to measure fat deposition and ROS production, respectively (12).

#### Transmission electron microscopy

Representative kidney sections were fixed in Trump’s fixative solution (4% formaldehyde and 0.1% glutaraldehyde in 0.1 M phosphate buffer) at room temperature overnight. Sections were then stained with aqueous uranyl acetate and lead citrate at the Mayo Clinic’s electron microscopy core facility. Micrographs focusing on mitochondria were taken using transmission electron microscopy (TEM, JEOL 1400 TEM, Peabody, MA), and mitochondrial morphology (e.g., cristae membrane and outer membrane architecture) examined in a random blinded fashion (31).

#### LPO measurement

C11 BODIPY 581/591 staining (Thermo Fisher Scientific) was performed to quantify LPO levels in the kidney tissue. The frozen sections were incubated with 2 μM BODIPY for 30 min at room temperature, protected from light, and then washed with PBS ×3. Subsequently, sections were stained with Hoechst nuclear stain (Thermo Fisher Scientific) for 10 min. Oxidation of the polyunsaturated butadienyl portion of C11-BODIPY leads to a shift of the fluorescence emission peak from ∼590 nm to ∼510 nm proportional to LPO. Images were taken under 594 nm and 488 nm under microscopes.

#### Malondialdehyde assay

Malondialdehyde (MDA), the end-product of LPO, was determined using a kit (Sigma-Aldrich, St. Louis, MO) following the manufacturer’s instructions. Briefly, 10 mg kidney tissue was homogenized with 300 μl MDA lysis buffer and centrifuged to get the supernatant, which was then incubated with thiobarbituric acid solution to form the MDA-TBA adduct. The adducts were read in a 96-well plate at 532 nm.

#### Ferroptosis markers

Because ferroptosis is an iron-dependent form of cell death, we first tested the iron level in kidney tissue (Sigma). Briefly, 10 mg kidney tissue was homogenized with the buffer, followed by centrifugation to obtain soluble material of which 50 μl was incubated with an iron assay buffer or iron reducer at 25°C for 30 min in the dark. Next, 100 μl iron probe was added to each well and incubated for 1 h, and finally the absorbance was measured at 593 nm.

To detect ferroptosis mediators, mRNA expression of *Gpx4* (Ss03384646, Thermo Fisher Scientific) and *Acsl4* (Ss03379974) was studied using quantitative real-time PCR in kidney tissues (32). *Gapdh* (Ss03375435) probes were applied in the Taqman assay. Fold-change of gene expression was calculated by the 2^-ΔΔct^ method.

#### ER stress

ER was extracted from kidney tissue using an ER enrichment kit (Novus Biologicals, Centennial, CO), and CHOP (Novus Biologicals), GRP94 (Abcam), and GAPDH (Abcam) protein expression was tested by Western blot (32).

### In vitro studies

#### Cell culture

Pig kidney epithelial cells (LLC-PK1, ATCC, Manassas, VA) were incubated with vehicle (2% EtOH and 20% BSA in PBS, control), PA (33) (0.5 mM in vehicle, Sigma), oxidized-LDL (34) (ox-LDL, 15 μg/ml in vehicle, Sigma), or their combination (PA + ox-LDL) for 24 h in an attempt to mimic the in vivo microenvironments of ND, HFD, PCSK9-GOF/ND, and PCSK9-GOF/HFD, respectively. In parallel PK1 cells, the ferroptosis inhibitor ferrostatin-1 (Fer-1, 1 μM in DMSO, Sigma) was also added to form four additional treatment groups: Fer-1+vehicle, Fer-1+PA, Fer-1+ox-LDL, and Fer-1+PA+ox-LDL. Subsequently, assays were performed on these eight groups of cells. To appraise the toxicity of PA, ox-LDL and Fer-1, PK1 were seeded in a 24-well plate (10^5^ cells/well) and immediately placed in a cytation-5 instrument (BioTek, Agilent Technologies, Pittsburgh, PA) at 37°C with 5% CO_2_ for 60 h. Proliferation was indexed by cell confluency.

#### Mitochondrial function

MitoSOX red reagent (2 μM, Thermo Fisher Scientific) and tetramethylrhodamine ethyl ester (50 nM, Thermo Fisher Scientific) were utilized to appraise mitochondrial ROS production and membrane potential, respectively (35). The average optical density and mean fluorescent intensity of each experimental group were normalized to that of the control group. Intracellular ATP level was measured using the ATPlite Luminescence Assay System (PerkinElmer, Waltham, MA). PK1 cells lysed by the mammalian cell lysis solution, which stabilizes the ATP, were incubated with substrate solution for 5 min, followed by 10 min dark adaption and luminescence measurement.

#### GSH assay

The intracellular levels of a non-enzymatic antioxidant were detected by the GSH assay kit (Sigma). The PK1 cell pellets (10^6^ cells/group) were homogenized by lysis buffer containing and centrifuged. Then, meta-phosphoric acid solution was added into each supernatant to deproteinate, followed by incubation with assay buffer and measurement of the absorbance at 412 nm at 0 min and 10 min.

#### Fe^2+^ in cells

To detect intracellular Fe^2+^, FerroOrange (Dojindo, Rockville, MD) was used. The cells were stained with a final concentration of 1 μM FerroOrange for 30 min at 37°C, washed with PBS, and live-cell images were immediately acquired under a fluorescence microscope from ten randomly selected regions of interest. The fluorescent intensity was measured by Fiji (1.53q, Wayne Rasband and contributors, NIH) and mean fluorescent intensity of each group was normalized to that of the control group.

#### Lipid deposit and LPO in vitro

ORO staining was performed to evaluate fat deposit in the paraformaldehyde-fixed PK1 cells as described above. Live PK1 cells in 6-well plates were digested by trypsin, collected into centrifuge tubes, and treated with 2 μM BODIPY probe for 20 min at 37°C in the dark. After washing with PBS, the staining fluorescence intensity was measured by flow cytometry (FACSCanto X SORP, BD Biosciences, San Jose, CA).

### Statistical analysis

GraphPad Prism (version 9.0.1, San Diego, CA) was applied for data analysis and graphs production. Normality distribution was tested first. Normally distributed data are shown as mean ± SD, while non-normally distributed data are expressed as median and interquartile range. Two-group comparisons used unpaired parametric or non-parametric tests as applicable. For comparison among multiple groups, one-way ANOVA was followed by post hoc Bonferroni if normally distributed or Dunn’s if non-normally distributed. To assess interactions among time points and multiple groups, two-way ANOVA followed by a Bonferroni adjustment was used. *P* < 0.05 indicated significance.

## Results

### In vivo and ex vivo studies

**Fig. 1:**
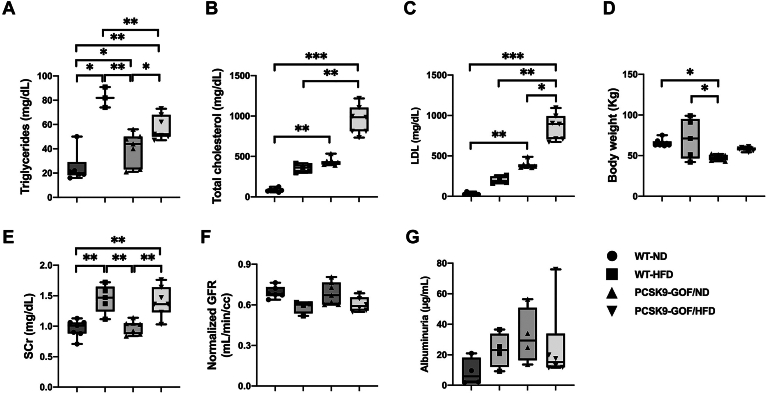
Lipid profile and kidney function in WT and proprotein convertase subtilisin/kexin type-9 (PCSK9)-gain-of-function (GOF) Ossabaw pigs fed with a 6-month normal diet (ND) or high-fat (HFD) diet. A: Triglycerides. B: Total cholesterol. C: LDL. D: Body weight. E: Serum creatinine (SCr). F: Glomerular filtration rate (GFR) per unit kidney volume. G: Albuminuria. ∗*P* < 0.05, ∗∗*P* < 0.01, and ∗∗∗*P* < 0.001 between groups. WT, wild-type.

Compared to WT-ND, all dyslipidemic experimental groups showed elevated TG levels, which was highest in the WT-HFD pigs (*P* < 0.05) (Fig. 1A). However, the two PCSK9-GOF groups had significantly higher total cholesterol levels than WT-ND (*P* < 0.01), with PCSK9-GOF/HFD higher than WT-HFD (*P* = 0.006). Total cholesterol levels in WT-HFD tended to be elevated as well (*P* = 0.09 vs. WT-ND) (Fig. 1B). LDL showed a similar pattern to total cholesterol (Fig. 1C). The PCSK9-GOF/ND pigs had lower body weight than both WT groups (*P* < 0.05, Fig. 1D). In terms of renal function, HFD showed elevated SCr levels compared with ND pigs (*P* < 0.01, Fig. 1E), while their GFR showed only a statistical trend for a difference among the groups (*P* = 0.08, Fig. 1F). There was no significant difference in albuminuria among the groups (Fig. 1G).

### Renal lipid handling

**Fig. 2:**
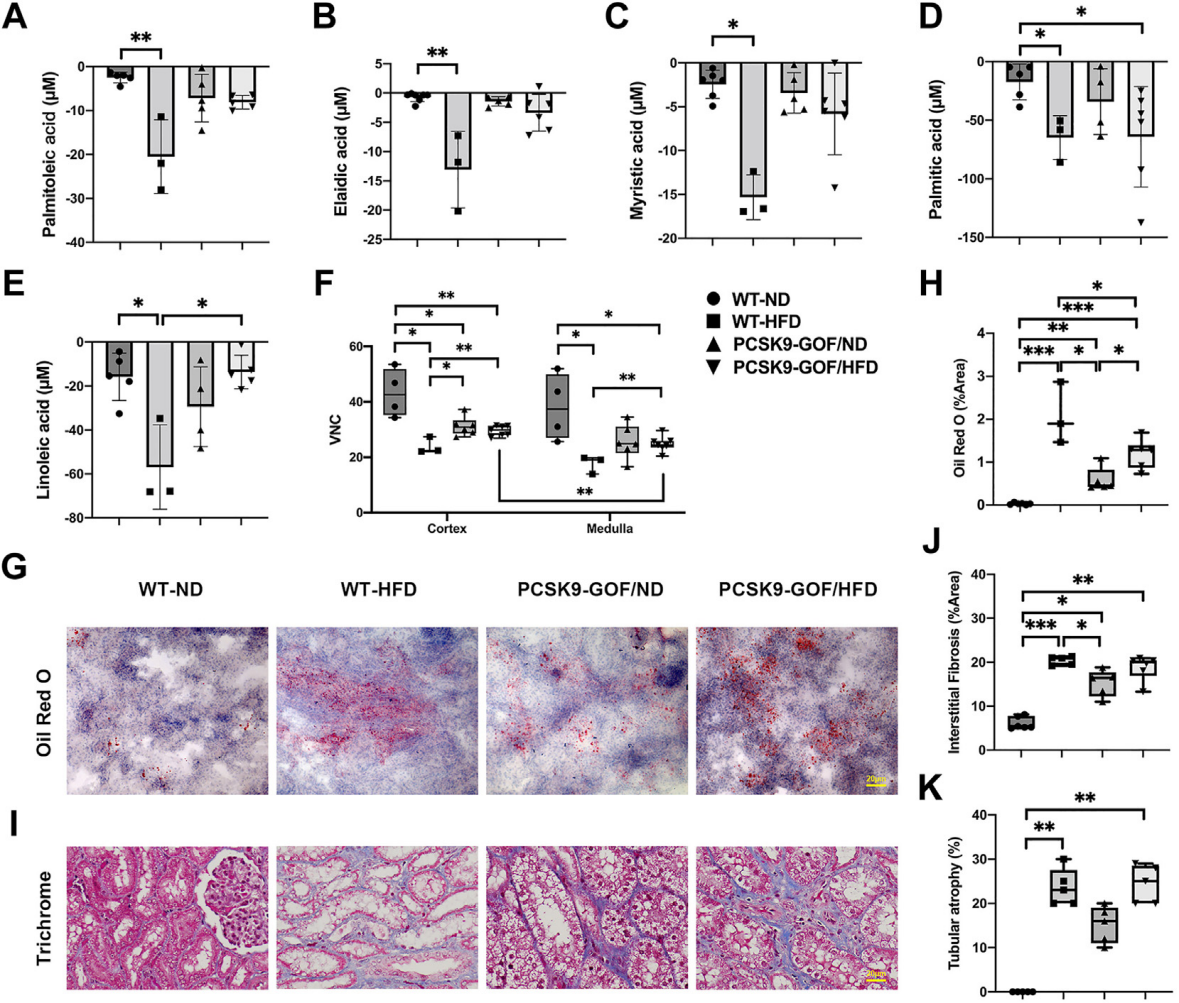
Renal fat retention, deposition, and fibrosis. A–E: FFA gradients across the kidneys. F: virtual-noncontrast (VNC) values obtained by CT, where lower values indicate fat accumulation. G–H: Increased Oil-red-O (ORO) staining showed that both HFD and PCSK9-GOF promoted fat deposit in the kidney, which was highest in WT-HFD. I–K: In Masson’s Trichrome staining, both HFD groups showed interstitial fibrosis and tubular atrophy, whereas PCSK9-GOF showed only interstitial fibrosis. Scale bar = 20 μm. ∗*P* < 0.05, ∗∗*P* < 0.01, and ∗∗∗*P* < 0.001 between groups. GOF, gain of function; HFD, high-fat diet; PCSK9, proprotein convertase subtilisin/kexin type-9. WT, wild-type.

The FFA gradients across the kidneys showed that two SFAs (myristic acid and PA), one MUFA (palmitoleic acid), one PUFA (linoleic acid), and one TFA (elaidic acid) were retained in WT-HFD kidneys, and PA was also highly retained by PCSK9-GOF/HFD (Fig. 2A–E). The lowest MDCT-VNC attenuation also suggested that WT-HFD developed fatty kidneys at 6 months (Fig. 2F). The two PCSK9-GOF groups also had similarly lower attenuation than WT-ND (indicating tissue lipid deposits), yet higher than in WT-HFD. Overall, the cortex and medulla showed similar VNC attenuation patterns, except that medullary attenuation in PCSK9-GOF/HFD was lower than its cortex (*P* = 0.0027). ORO staining confirmed that the greatest fat deposits occurred in WT-HFD kidneys but were also elevated in the two PCSK9-GOF groups (*P* < 0.01 vs. WT-ND) (Fig. 2G, H). PCSK9-GOF/HFD had higher lipid deposition than PCSK9-GOF/ND kidneys.

**Supplementary Fig S1:**
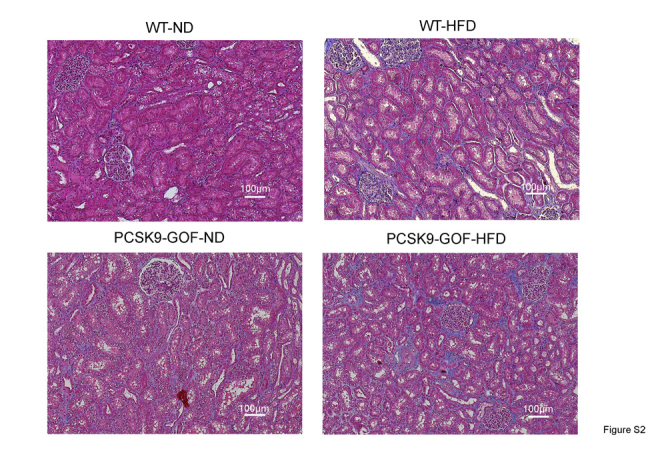
Lower magnification (10×) images of Masson’s trichrome staining in each group, showing elevated interstitial fibrosis in the experimental groups compared to WT-ND.

Masson’s Trichrome staining showed significantly greater IF and TA characterized by the thinning of tubular epithelial cells surrounded by collagen deposition in WT-HFD kidneys (*P* = 0.0006 and *P* = 0.0021, respectively) and PCSK9-GOF/HFD (*P* = 0.0045 and *P* = 0.0021, respectively). PCSK9-GOF/ND (*P* = 0.0472) developed only IF (Fig. 2I–K and Supplemental Fig. S1).

### Kidney mitochondrial abnormalities in dyslipidemic pigs

**Fig. 3:**
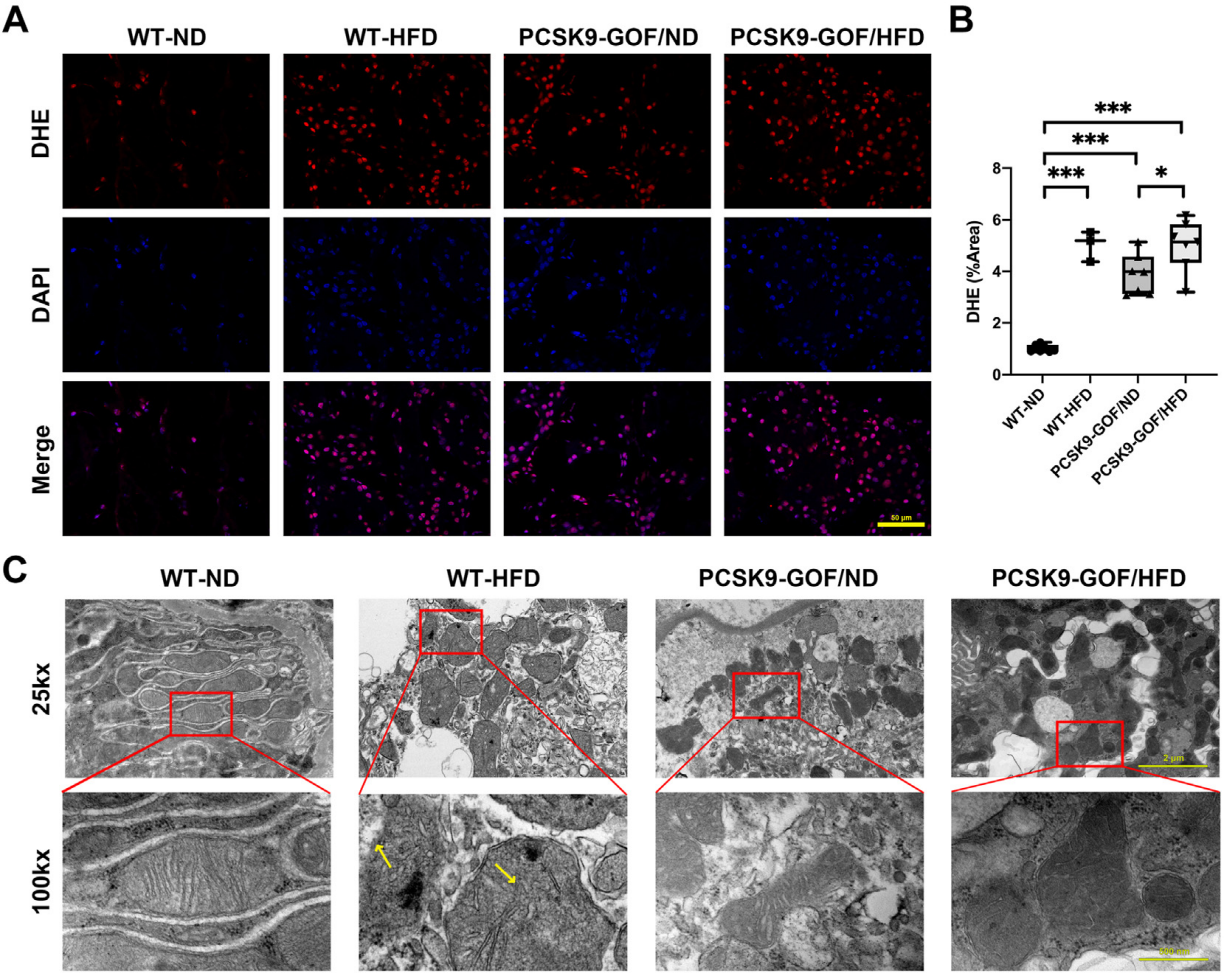
Kidney mitochondria dysfunction in pigs with dyslipidemia. A, B: Dihydroethidium (DHE) staining showed that both HFD and PCSK9-GOF enhanced reactive oxygen species production in the kidneys. C: Representative transmission electron microscope images of kidney sections showed mitochondria morphological changes in dyslipidemia groups, especially in WT-ND: shrunken mitochondria, decline of mitochondria cristae, and rupture of outer mitochondria membrane. Scale bar = 50 μm for DHE. Scale bar = 2 μm and 500 nm for TEM. ∗*P* < 0.05 and ∗∗∗*P* < 0.001 between groups. GOF, gain of function; HFD, high-fat diet; ND, normal diet; PCSK9, proprotein convertase subtilisin/kexin type-9; TEM, transmission electron microscopy. WT, wild-type.

WT-HFD and the two PCSK9-GOF kidneys all showed elevated dihydroethidium staining (*P* < 0.001 vs. WT-ND) (Fig. 3A, B). Furthermore, dyslipidemia induced mitochondrial morphological changes. Representative TEM images showed in pigs with dyslipidemia, especially WT-HFD, shrunken mitochondria, decline of mitochondria crista, and rupture of the outer mitochondrial membrane (Fig. 3C).

### HFD-induced kidney ferroptosis

**Fig. 4:**
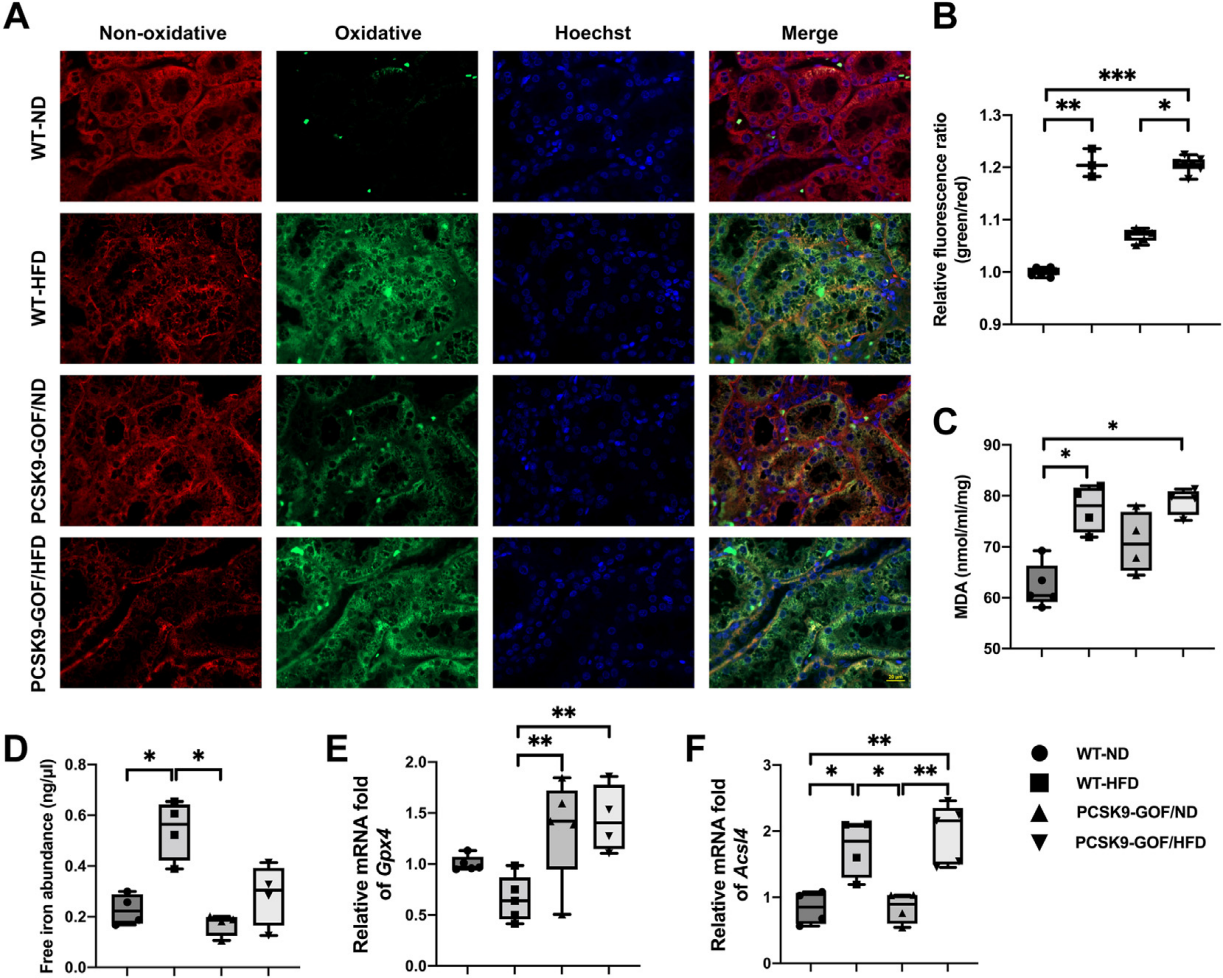
High-fat diet induced ferroptosis in the kidney. A, B: Representative C11-BODIPY 581/591 staining showed increased renal lipid peroxidation in the two HFD groups. C: Malondialdehyde (MDA). D: Ferrous ions increased in WT-HFD. E, F: Ferroptosis markers (*Gpx4* and *Acsl4*) mRNA expression in kidney tissue. Scale bar = 20 μm. ∗*P* < 0.05, ∗∗*P* < 0.01, and ∗∗∗*P* < 0.001 between groups. *Acsl4*, acyl-CoA synthetase long-chain family member-4; *Gpx4*, glutathione peroxidase-4; WT, wild-type.

Given that the kidneys in pigs with dyslipidemia showed oxidative stress and retained excessive PUFAs, we hypothesized that they developed LPO. Using the C11-BODIPY 581/591 probe, we found that the ratio of oxidative to non-oxidative lipids was remarkably increased in the two HFD groups compared with WT-ND, primarily in the renal tubules (Fig. 4A, B) and in PCSK9-GOF/HFD versus PCSK9-GOF/ND group (*P* = 0.032), which was not different from WT-ND (*P* = 0.086). The level of the LPO product MDA was elevated in the HFD pigs, regardless of their genetic background (*P* < 0.05 versus WT-ND) (Fig. 4C).

The labile iron pool (intracellular ferrous ions, Fe^2+^) in fresh kidney tissue increased in the WT-HFD compared with both WT-ND (*P* = 0.021) and PCSK9-GOF/ND (*P* = 0.004), but was not different from PCSK9-GOF/HFD (*P* = 0.271) (Fig. 4D). WT-HFD kidneys showed downregulated mRNA expression of *Gpx4*, which counteracts ferroptosis, compared with the two PCSK9-GOF groups (*P* < 0.01), and also strongly tended to be lower than WT-ND (*P* = 0.056) (Fig. 4E). Contrarily, *Acsl4* mRNA expression was increased in the HFD compared with ND (Fig. 4F).

### PCSK9-GOF induced kidney ER stress

**Fig. 5:**
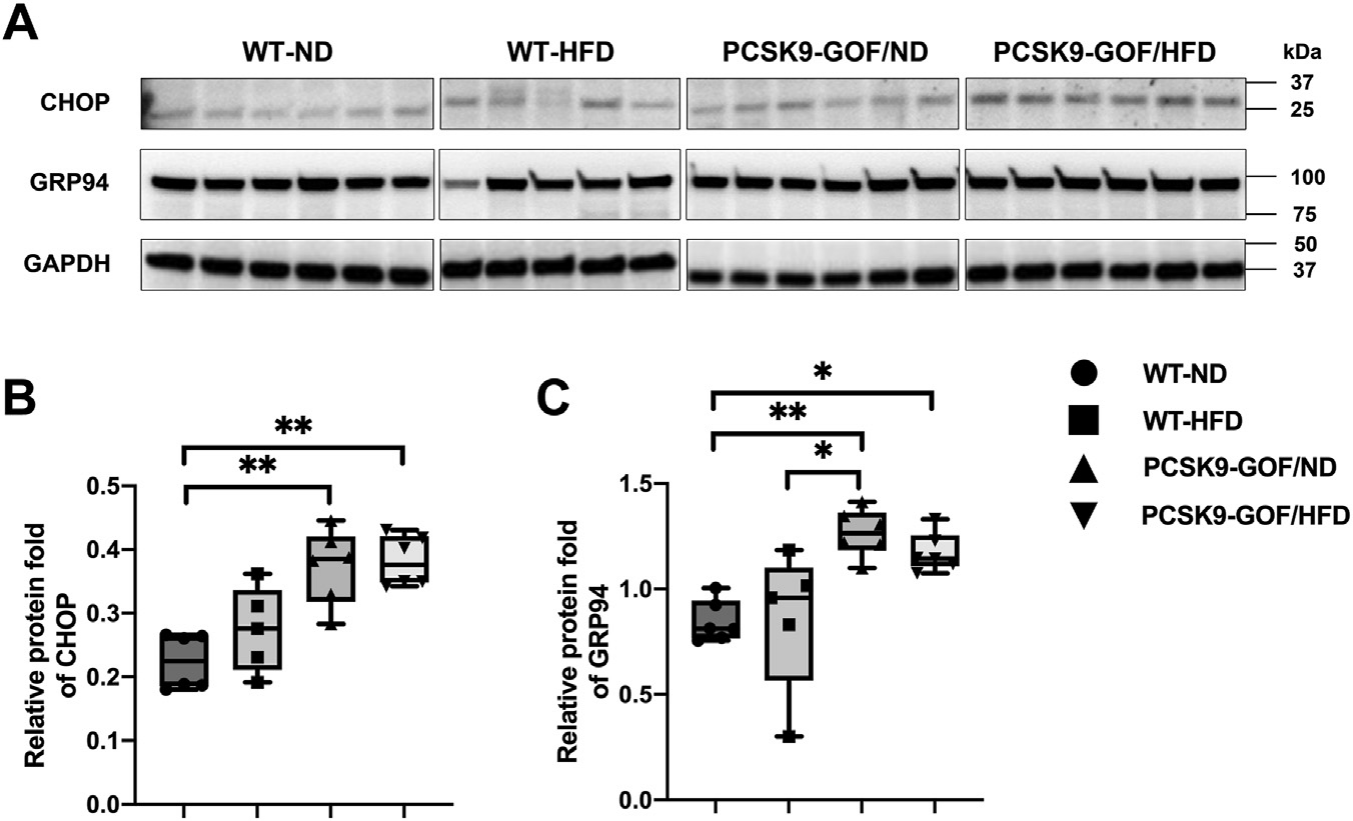
PCSK9-GOF induces kidney endoplasmic reticulum stress. A–C: Western blot showed upregulated CHOP and GRP94 protein expression in mitochondria isolated from kidneys of the two PCSK9-GOF groups. ∗*P* < 0.05 and ∗∗*P* < 0.01 between groups. GOF, gain of function; PCSK9, proprotein convertase subtilisin/kexin type-9; WT, wild-type.

Both HFD and PCSK9-GOF induced oxidative stress, whereas ferroptosis was more closely linked to HFD. On the other hand, in isolated ER the expression of both CHOP and GRP94 was significantly increased in the two PCSK9-GOF groups compared with WT-ND (*P* < 0.01, Fig. 5A–C), suggesting ER stress.

### In vitro studies

#### Fer-1 rescued PA-induced ferroptosis in PK1 cells

**Supplementary Fig S2:**
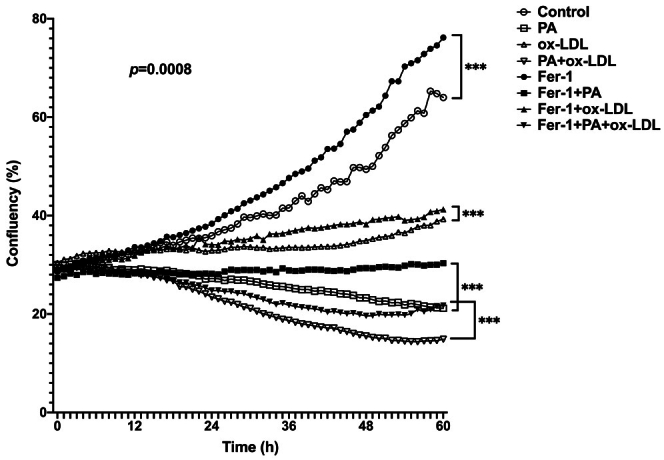
PA inhibited and ox-LDL attenuated the proliferation of PK1 cells, which Fer-1 treatment partly rescued. ∗∗∗*P* < 0.001 between groups.

The proliferation of PK1 cells incubated with PA alone (*P* < 0.001) and PA+ox-LDL (*P* < 0.001) was markedly inhibited, while the ox-LDL group was significantly but slightly less attenuated (*P* < 0.001 vs. control) (Supplemental Fig. S2). However, Fer-1 treatment increased although not normalized proliferation inhibition in each lipid-treated group (*P* < 0.001 vs. Fer-1–untreated cells).

**Fig. 6:**
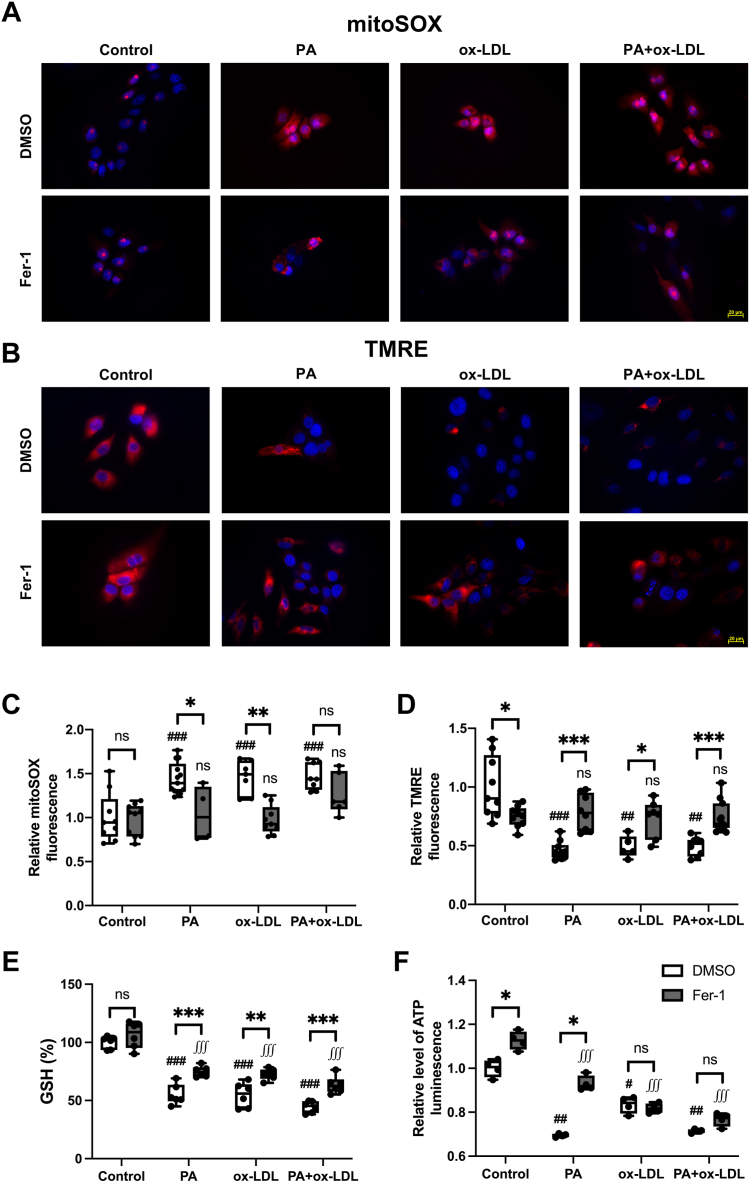
Ferrostatin-1 rescued palmitic acid and oxidized-LDL–induced mitochondrial damage in PK1 cells. A and C: Mito-SOX staining showed elevated reactive oxygen species production in PA, ox-LDL, and PA + ox-LDL cells, which Fer-1 reversed. B and D: Tetramethylrhodamine ethyl ester (TMRE) staining showed decreased mitochondrial membrane potential in the PA, ox-LDL, and PA + ox-LDL groups, which Fer-1 recovered. E: PA, ox-LDL, and PA + ox-LDL treatment significantly decreased GSH level in PK1 cells, which Fer-1 improved. F: PA, ox-LDL, and PA + ox-LDL treatment decreased ATP production in PK1 cells, and Fer-1 only ameliorated PA-mediated ATP decrease. ns: no significance, ∗*P* < 0.05, ∗∗*P* < 0.01, and ∗∗∗*P* < 0.001 between groups; ^#^*P* < 0.05, ^##^*P* < 0.01, and ^###^*P* < 0.001 over graph bars compared with DMSO group; ^∭^*P* < 0.001 over graph bars compared with Fer-1 group. PK1, pig kidney epithelial cell.

Assessing mitochondrial integrity, the mitoSOX fluorescence increased in all lipid-treated cells (*P* < 0.001 vs. control, Fig. 6A, C), whereas tetramethylrhodamine ethyl ester decreased (*P* < 0.01) (Fig. 6B, D), indicating mitochondrial damage. However, Fer-1 consistently protected the cells from mitochondrial damage. All treated PK1 cells manifested a fall in GSH (*P* < 0.001 vs. control), which was markedly elevated by Fer-1 treatment (Fig. 6E, *P* < 0.01 vs. baseline), although they remained lower than control (*P* < 0.001). Moreover, intracellular ATP levels fell in all lipid-treated PK1 compared with control cells (Fig. 6F). Fer-1 treatment rescued only the PA-mediated decrease (*P* = 0.029).

**Fig. 7:**
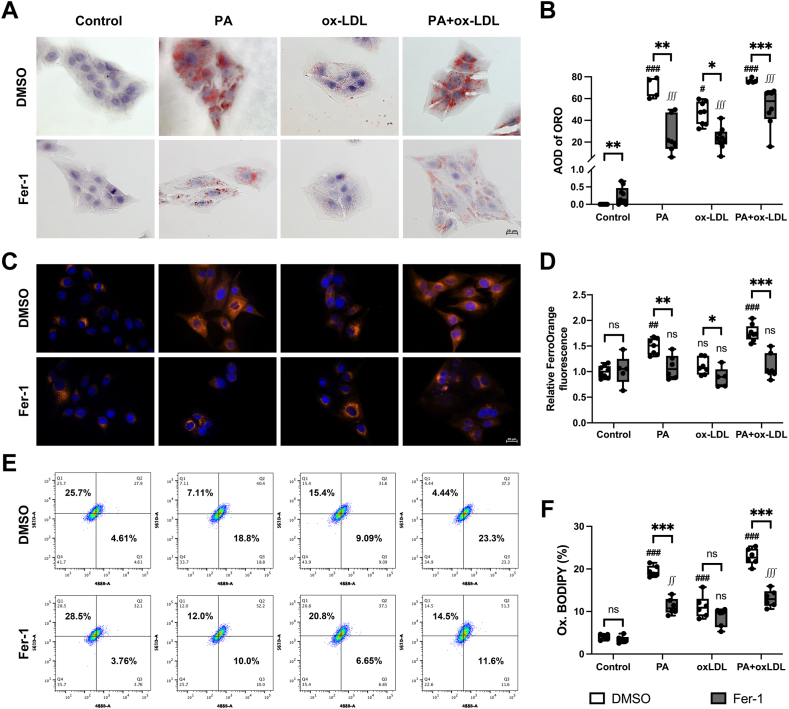
Ferrostatin-1 rescued palmitic acid–induced ferroptosis in PK1 cells. A, B: Oil-red-O staining showed increased intracellular lipid droplets in the PA, ox-LDL, and PA + ox-LDL groups, which Fer-1 significantly blunted. C, D: FerroOrange staining detected increased intracellular ferrous ions level in the PA-treated and PA + ox-LDL–treated PK1 cells. E, F: Flow cytometry using C11-BODIPY 581/591 probe showed that PA, ox-LDL, and PA + ox-LDL treatment increased oxidized lipids in PK1 cells, which Fer-1 decreased. Scale bar = 20 μm ns: no significance, ∗*P* < 0.05, ∗∗*P* < 0.01, and ∗∗∗*P* < 0.001 between groups; ^#^*P* < 0.05, ^##^*P* < 0.01, and ^###^*P* < 0.001 over graph bars compared with DMSO group; ^∬^*P* < 0.01, ^∭^*P* < 0.001 over graph bars compared with Fer-1 group. Ox-LDL, oxidized-LDL; PK1, pig kidney epithelial cell.

PA and ox-LDL cells also elicited prominent intracellular lipid droplets (ORO staining), which Fer-1 reduced (*P* < 0.05) (Fig. 7A, B). PA and PA + ox-LDL, but not ox-LDL alone, also magnified iron content in PK1 compared with control cells, which was markedly decreased by Fer-1 (Fig. 7C, D). Flow cytometry using C11-BODIPY 581/591 in PK1 cells showed remarkably increased LPO after incubation with PA, ox-LDL, and their combination, which Fer-1 reversed only in cells incubated with PA or PA+ox-LDL (Fig. 7E, F).

## Discussion

The present study shows that dyslipidemia induced by either HFD or PCSK9-GOF elicits renal fat deposition, dysfunction, and fibrosis. However, the severity and type of dyslipidemia were linked to activation of slightly different mechanisms of kidney injury. A 6-month HFD was dominated by hypertriglyceridemia and induced kidney ferroptosis, while PCSK9-GOF caused primarily hypercholesterolemia and ER stress.

We studied PCSK9-GOF pigs, a well-established swine model of hypercholesterolemia, to evaluate target-organ effects of dyslipidemia. Importantly, PCSK9 inhibition is effective in patients with various forms of dyslipidemia (36), underscoring the ubiquitous role of PCSK9 in hypercholesterolemia. Therefore, our model is relevant to assess the effect of the hyperlipidemic milieu on renal function. The direct role of hypercholesterolemia in prompting renal fibrosis and disease progression in the clinical setting is unclear. While lowering LDL-C reduces the risk of atherosclerotic events in CKD, it does not necessarily slow kidney disease progression, (37) possibly because many patients have underlying CKD due to other etiologies. Indeed, dyslipidemia may serve as a “second hit” to magnify the vulnerability of the kidney to additional insults (38), so that lowering LDL alone may not restore renal function. Contrarily, the role of hypertriglyceridemia in inducing renal fibrosis is relatively clear. (39).

Interestingly, we observed elevated SCr in HFD-fed pigs but only a statistical trend toward differences in GFR among the groups. While GFR is superior to SCr alone for assessing kidney function, in CKDs, GFR often declines more gradually, reflecting ongoing damage over time. On the other hand, while SCr is a useful marker of kidney function, it can be influenced by various factors such as muscle mass, diet, and hydration status that do not directly reflect the actual filtration capacity of the kidneys. Therefore, SCr and GFR measurements are often considered together to assess kidney function, along with clinical context. Notably, the fibrosis and inflammation observed in our study may eventually lead to a significant decline in GFR, as shown in a previous study (40).

We found increased renal fat deposits in dyslipidemic pig kidneys and in vitro PA and ox-LDL both elicited accumulation of intracellular lipids. Lipid droplets are commonly recognized in renal tubular cells of dyslipidemic or obese subjects. They may be cytoprotective, being cellular energy stores engaged in biological processes, particularly under conditions of nutrient stress or increased energy demand. (41) However, during an imbalance between lipid storage and metabolism, such as impaired FA oxidation, lipid metabolites may become deleterious, leading to lipotoxicity. The accumulation of toxic lipid metabolites might contribute to development of renal fibrosis (42), although the contribution of lipid droplets per se to renal fibrosis has not been fully established.

Dyslipidemia-induced renal injury attributes to diverse injury mechanisms such as apoptosis (43) and autophagy (44), but its effect on ferroptosis in the fibrotic kidneys has not been elucidated. Ferroptosis represents an iron-dependent oxidative form of cell death that is associated with increased LPO (45). In the present study, we observed ferroptosis in WT-HFD pig kidneys. Inhibition of ferroptosis in vitro by Fer-1 blunted the damaging effect of lipotoxicity on PK1 cells and partly recovered antioxidant capacity. Kidney cells, particularly the tubular epithelium, are abundant with mitochondria, which utilize FFAs for β-oxidation as the primary source of ATP production (46). However, excessive influx of FFAs overwhelms the oxidative capacity, leading to harmful ROS formation and renal fibrosis (47, 48). Indeed, HFD kidneys retained FFA, and PK1 cells exposed to PA or ox-LDL showed mitochondrial dysfunction denoted by increased ROS generation, decreased membrane potential, and declined ATP production.

While indispensable for metabolic and physiological activities (49), excess iron might promote the production of hydroxyl radicals, LPO, oxidative stress, and cell damage and is associated with mitochondrial dysfunction. Oxidative lipid species are often detoxified by GSH and GPX4, the deficiency of which is linked to ferroptosis (50). Indeed, *Gpx4* in WT-HFD tended to be lower than in WT-ND and was significantly lower than in both PCSK9-GOF groups and PA-treated PK1 cells showed decreased GSH. Therefore, iron overload and downregulated expression of GPX4 and GSH limited the ability to curtail ROS, enhancing cellular sensitivity to ferroptosis.

We also observed elevated *Acsl4* mRNA expression in HFD. The ACSLs, important enzymes in the metabolism of long-chain FA, participate in lipid droplet biogenesis (51). During ferroptosis, ACSL4 ligates free PUFAs with CoA for their subsequent incorporation into phospholipids, substrates for LPO (52, 53). Interestingly, we found not only a PUFA but also a MUFA, a TFA, and two SFAs retained in WT-HFD kidneys, while only a SFA retained in the PCSK-9-GOF/HFD kidneys. The excessively retained PUFA in WT-HFD may explain why ferroptosis was more linked to TGs (which were particularly elevated in HFD) than LDL-C, and impaired renal function.

The causal relationship between ferroptosis and renal fibrogenesis is still unclear. It is implicated in fibrosis through the generation of ROS and subsequent LPO, fostering a microenvironment conducive to fibroblast activation and extracellular matrix deposition. The process interlinks with inflammation, whose mediators facilitate ferroptosis by modulating iron metabolism and antioxidant responses, while ferroptosis in turn amplifies inflammation via damage-associated molecular patterns release and immune cell activation. (54) While ferroptosis exacerbates acute and CKDs (55), it may also contrarily ameliorate fibrosis (56).

Notably, unlike WT-HFD, PCSK9-GOF induced ER stress rather than ferroptosis. PCSK9 increases the degradation of the LDL-receptor and reduces LDL-C clearance, causing hypercholesterolemia (57). Accumulating studies underscore the relationship between PCSK9 and CKD (58). ER plays a crucial role in controlling circulating lipid levels, and the ER-resident chaperone GRP94 protects the LDL-receptor from PCSK9-induced degradation (59). When the cholesterol levels overwhelm the ER, like in PCSK9-GOF, a shift of excessive cholesterol into the ER induces stress and misfolded proteins (60), which may in turn induces insulin resistance, inflammation, and kidney damage. ER stress triggers fibrosis through the unfolded protein response, contributing to inflammation and augmenting myofibroblast fibrogenic activity. (61) Notably, a synergistic crosstalk exists between ferroptosis and ER stress, wherein the ER stress byproduct ROS may aggravate ferroptosis. (62, 63) This interaction could perpetuate a vicious cycle that promotes fibrosis, suggesting a complex relationship between ferroptosis and ER stress.

Elucidating which lipid contributes to ferroptosis or ER stress may be help unravel their mechanisms. Ferroptosis may be related to PA and linoleic acid (Fig. 2D, E). (64, 65) Furthermore, PA was the only FA retained in PCSK9-GOF/HFD, suggesting that ER stress can be also induced by PA in vivo. Interestingly, PA may be associated with both ferroptosis and ER stress. (66) However, our in vitro study shows that PA induced ferroptosis but not ER stress. Our study showed that ox-LDL–induced oxidative stress, mitochondrial dysfunction, and LPO, which are all involved in the mechanisms of cell injury and death. Furthermore, hydroxynonenal, a major aldehyde product of LPO, induces apoptosis by targeting ER stress pathways and mitochondrial dysfunction (67), demonstrating a direct link between ox-LDL and ER stress.

Our preclinical animal study revealed that diverse types of dyslipidemia caused renal damage by different types of cell injury or death. However, our study faces some limitations. We focused on dyslipidemia rather than other metabolic parameters linked to renal injury. The lower body weight in PCSK9-GOF/ND versus WT pigs probably resulted from being the 1^st^ generation after cloning (12). Fer-1 was not applied in vivo to enhance the evidence of ferroptosis in WT-HFD kidneys, an impractical approach in large animals. In addition, in vitro models infrequently fully replicate the in vivo model, which might explain why PA + ox-LDL also increased intracellular iron in PK1 cells unlike in PCSK9-GOF/HFD in vivo. Finally, the underlying signaling pathways for ferroptosis and ER stress also remain to be elucidated.

In conclusion, the current study shows that while both HFD and PCSK9-GOF cause dyslipidemia, leading to renal fibrosis, the underlying mechanisms might differ. Furthermore, the development of ferroptosis versus ER stress might depend on the type or the severity of dyslipidemia. These observations support the application of targeted interventions to blunt renal lipotoxicity consistent with the instigating factor.

## Data availability

The raw data of this study are available from the corresponding author upon reasonable request.

## Supplemental data

This article contains supplemental data.

## Conflict of interest

Dr Lerman is an advisor to CureSpec. The other authors declare that they have no conflicts of interest with the contents of this article.
